# Cu_2_O nanoparticles electrodeposited on carboxylated multiwalled carbon nanotubes for sensitive electrochemical detection of nitrate in seawater

**DOI:** 10.3389/fchem.2026.1947669

**Published:** 2026-08-31

**Authors:** Yekai Shao, Dewang Li, Xiaobo Ni, Mingxuan Li, Jianhang Yue, Feng Zhou, Jiangwen Zhao

**Affiliations:** 1 State Key Laboratory of Satellite Ocean Environment Dynamics, Second Institute of Oceanography, Ministry of Natural Resources, Hangzhou, China; 2 School of Chemistry and Chemical Engineering, Zhejiang Sci-Tech University, Hangzhou, China; 3 Key Laboratory of Marine Ecosystem Dynamics, Second Institute of Oceanography, Ministry of Natural Resources, Hangzhou, China; 4 Zhuji Cotex Electricity Technology Co., Ltd., Zhuji, China

**Keywords:** carboxylated multiwalled carbon nanotubes, cuprous oxide, electrochemical sensing, nitrate determination, seawater

## Abstract

Rapid and sensitive determination of nitrate (NO_3_^−^) is crucial as NO_3_^−^ contamination in aquatic environments poses increasing risks to ecosystem stability and drinking water safety. Herein, an electrochemical NO_3_^−^ sensor was reported based on cuprous oxide (Cu_2_O) nanoparticles integrated with carboxylated multiwalled carbon nanotubes (cMWCNTs) on indium tin oxide (ITO) electrode. The sensing interface was constructed by drop-casting conductive cMWCNTs network followed by *in situ* electrodeposition of Cu_2_O nanoparticles. The cMWCNT scaffold improved interfacial charge transport, enlarged the accessible surface area, and promoted the homogeneous distribution of Cu_2_O, while Cu_2_O acted as nanocatalyst for NO_3_^−^ electroreduction. Electrochemical characterization confirmed that Cu_2_O@cMWCNTs/ITO electrode exhibited lower charge-transfer resistance and stronger NO_3_^−^ reduction current than Cu_2_O modified ITO electrode. Under optimized conditions, the fabricated sensor enabled NO_3_^−^ detection over two linear ranges of 1–100 and 100–800 μM, with limit of detection of 0.79 μM. The sensor showed good tolerance toward dissolved oxygen and common inorganic ions, as well as satisfactory fabrication reproducibility. NO_3_^−^ detection in seawater was also achieved. This simple and effective Cu_2_O-carbon nanotube interface showed potential for electrochemical monitoring of NO_3_^−^ in environmental waters.

## 1 Introduction

Nitrate (NO_3_^−^) is an abundant inorganic nitrogen species in surface water, groundwater, and coastal seawater (Singh et al., 2019). Its concentration in aquatic environments is strongly influenced by fertilizer runoff, animal waste, municipal wastewater, and industrial discharge (Kudr et al., 2017). In coastal regions, riverine input and land-based pollution can further increase NO_3_^−^ levels and change the nutrient balance of marine systems (Kundu et al., 2024). Elevated NO_3_^−^ concentrations may accelerate eutrophication and stimulate excessive algal growth, which can eventually deteriorate water quality and disturb aquatic ecosystems (Ajayi and Teepoo, 2024). From a public-health perspective, NO_3_^−^ can be converted to nitrite (NO_2_^−^) under certain biological conditions, leading to methemoglobinemia and the possible formation of N-nitrosamine compounds (Zhou et al., 2026). Therefore, rapid, reliable and sensitive determination of NO_3_^−^ in environmental waters is important for evaluating water quality.

Currently, spectrophotometric methods and ion chromatography are commonly used for NO_3_^−^ detection (Wang et al., 2013). Although these methods provide reliable analytical performance, they generally require large instrumentation, relatively complicated operation, and trained operators (Wang et al., 2017). These limitations hinder their application in rapid and high-frequency monitoring of complex environmental water samples. Electrochemical methods provide an alternative route for NO_3_^−^ detection because they use simple equipment, consume small sample volumes, and need short analysis time (Wu J. et al., 2026). The development of advanced electrochemical sensing platforms for NO_3_^−^ determination in water is highly desirable.

Nanomaterial modification has become an effective strategy for improving the analytical performance of electrochemical sensing interfaces (Fan et al., 2024; Gu et al., 2026; Huang et al., 2022; Luo et al., 2022). By tailoring the composition, morphology, and interfacial structure of electrode modifiers, nanomaterials can increase the number of accessible electroactive sites, facilitate charge transfer, and regulate the adsorption and conversion of target species (Huang et al., 2023; Ma et al., 2024; Yu et al., 2024). In electrochemical NO_3_^−^ sensing, the electrode interfacial materials play a decisive role in catalytic activity, electron-transfer efficiency, sensitivity, and operational stability (Bu et al., 2024). Copper-based materials are among the most widely used and representative electrocatalysts for NO_3_^−^ reduction (Wangchuk et al., 2025). This is mainly attributed to their appropriate adsorption affinity toward NO_3_^−^ and nitrate-reduction intermediates, which facilitates electron transfer during the reduction process while partially suppressing the competing hydrogen evolution reaction (Patella et al., 2021). In addition, the interaction between the Cu 3d electronic states and the antibonding orbitals associated with nitrate can help lower the energy barrier for NO_3_^−^ electroreduction (Aidli et al., 2024). As a typical copper-based semiconductor, cuprous oxide (Cu_2_O) has attracted considerable attention because of its low cost, facile preparation, and high electrocatalytic activity (Lu et al., 2023). It has been proven that Cu_2_O interfaces and oxygen vacancies generated during electrochemical reduction of Cu_2_O can promote the adsorption and transformation of NO_3_^−^ and related intermediates (Kumar et al., 2025). Thus, Cu_2_O has great potential for constructing high-performance interfaces for electrochemical detection of NO_3_^−^.

Despite its favorable electrocatalytic activity, Cu_2_O directly loaded on planar electrodes may suffer from limited active sites, nanoparticle aggregation, hindered interfacial charge transfer, and insufficient utilization of active components. Introducing conductive carbon nanomaterials as interfacial modifiers is effective for preparing Cu_2_O-based composite materials (Xia et al., 2022). Carbon-based nanomaterials [e.g., graphene (Deng et al., 2023; Han et al., 2022; Zhou et al., 2022a; Zou et al., 2022), carbon nanotubes (Zhong et al., 2026; Zhou et al., 2022b), and graphene quantum dots (Wang L. et al., 2025; Zhang C. et al., 2023)], can serve as catalyst supports, conductive enhancers, or active electrocatalysts to improve the performance of electrochemical sensors (Huang et al., 2024; Yan et al., 2025). For instance, multiwalled carbon nanotubes (MWCNTs) possess large specific surface area, chemical stability, and rapid electron-transport characteristics (Cui et al., 2023; Zheng et al., 2022). Thus, carbon nanotubes are useful Supplementary Material for constructing modified electrodes. They can also be employed as supporting frameworks for the loading and growth of nanomaterials. Compared with bare electrodes, MWCNT-modified layers increase the effective electroactive area, improve interfacial conductivity, and provide abundant nucleation and anchoring sites for metal or metal oxide nanoparticles, thereby enhancing the dispersion and utilization of active materials (Atta et al., 2021). Moreover, compared with single-walled carbon nanotubes, MWCNTs offer practical advantages such as lower cost, easier scale-up, better structural stability, and more convenient surface functionalization, making them promising for constructing low-cost and high-performance electrochemical NO_3_^−^ sensors. In particular, carboxylated multiwalled carbon nanotubes (cMWCNTs) contain abundant oxygen-containing functional groups, such as carboxyl and hydroxyl groups, which improve their aqueous dispersibility (Wang S. et al., 2025). A conductive cMWCNT network is therefore expected to mitigate sluggish charge transfer associated with Cu_2_O modification and promote uniform deposition of Cu_2_O nanoparticles.

Herein, Cu_2_O nanoparticle/carboxylated multiwalled carbon nanotube composite (Cu_2_O @cMWCNTs) sensing interface was developed for sensitive electrochemical detection of NO_3_^−^ in seawater. The sensing interface was fabricated by first drop-casting cMWCNTs onto ITO electrode to form conductive carbon nanotube network, followed by *in situ* electrodeposition of Cu_2_O nanoparticles on cMWCNT surface. This architecture integrated the electrocatalytic activity of Cu_2_O with the conductive scaffold effect of cMWCNTs, thereby facilitating interfacial electron transfer and improving Cu_2_O dispersion. The resulting Cu_2_O@cMWCNTs modified electrode exhibited enhanced electrochemical response toward NO_3_^−^. This work provides a simple and effective strategy for rapid electrochemical detection of NO_3_^−^ in environmental samples.

## 2 Materials and methods

### 2.1 Preparation of cMWCNTs and Cu_2_O@cMWCNTs modified electrodes

A homogeneous cMWCNT dispersion (0.05 mg/mL) was prepared by ultrasonication of cMWCNT powder for 4 h. The cMWCNTs exhibited favorable dispersibility in aqueous media. 50 μL of the well-dispersed cMWCNT suspension was drop-cast onto the cleaned ITO surface (0.5 cm × 5 cm) to cover the working area. The electrode was then dried under an infrared lamp for 30 min to evaporate the solvent and form a stable cMWCNT film. After cooling to room temperature, the resulting electrode was denoted as cMWCNTs/ITO. Cu_2_O was then prepared by electrodeposition. Briefly, 0.2 M copper tartrate solution was prepared by dissolving 1.501 g of L-tartaric acid and 2.497 g of CuSO_4_.5H_2_O in 50 mL of ultrapure water under continuous stirring. The pH of the solution was adjusted to pH 10 using NaOH (4 M). L-Tartaric acid served as a complexing agent for Cu^2+^ ions, thereby regulating the deposition behavior of copper species on the electrode surface and promoting the uniform formation of Cu_2_O. The electrodeposition was performed in 0.2 M copper tartrate solution at room temperature by applying a constant potential of −0.4 V vs. Ag/AgCl on cMWCNTs/ITO electrode for 30 s, allowing Cu_2_O nanoparticles to grow *in situ* on cMWCNT-modified surface. The obtained composite electrode was denoted as Cu_2_O@cMWCNTs/ITO. Electrochemical impedance spectroscopy (EIS) was employed to investigate the interface characteristics. EIS measurements were carried out in an electrolyte solution containing [Fe(CN)_6_]^3-/4-^ (0.5 mM) containing KCl (0.1 M) to evaluate the interfacial charge-transfer resistance of different modified electrodes. The frequencies range from 10^4^ to 10^−1^ Hz.

### 2.2 Electrochemical detection of NO_3_^−^

The as-prepared Cu_2_O@cMWCNTs/ITO electrode was directly used for the electrochemical detection of NO_3_^−^ without additional activation or further modification. 0.1 M Na_2_SO_4_ at pH 2 (adjusted with diluted H_2_SO_4_) was selected as the supporting electrolyte. Differential pulse voltammetry (DPV) was used to record the electrochemical responses of NO_3_^−^ solutions with different concentrations. The parameters included step of 0.004 V, amplitude of 0.05 V, pulse width of 0.2 s, sampling width of 0.0167 s, pulse period of 0.5 s. For real sample analysis, the seawater samples from Zhejiang coastal area (Zhoushan, China) were analyzed. The seawater was filtered through a 0.22 μm membrane filter and then acidified with diluted sulfuric acid to pH 2 before analysis.

## 3 Results and discussion

### 3.1 Construction of the Cu_2_O@cMWCNTs/ITO sensing interface and NO_3_^−^ detection strategy

**FIGURE 1:**
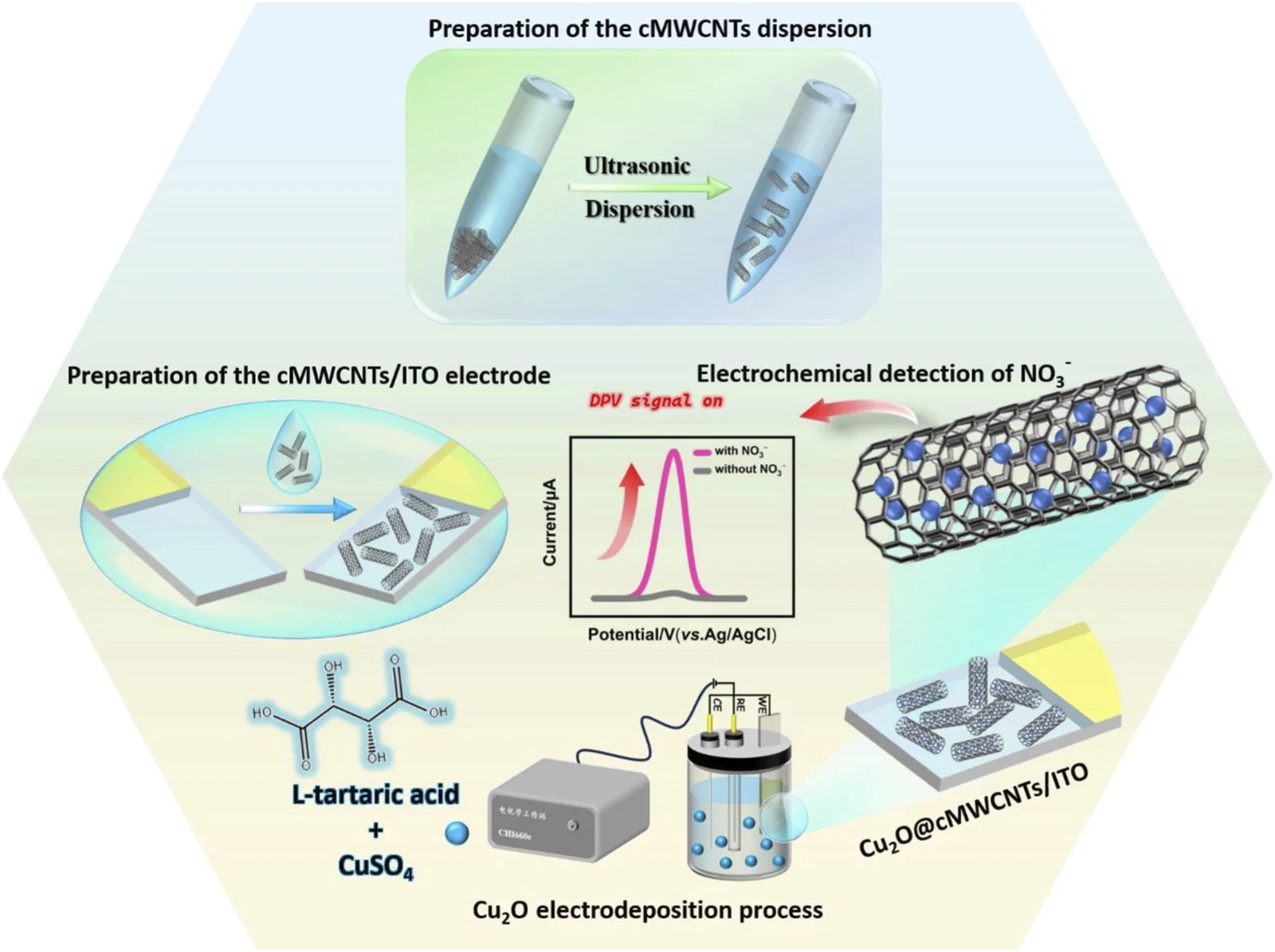
Schematic illustration of the fabrication of the Cu_2_O@cMWCNTs/ITO sensing interface and its application for electrochemical NO_3_^−^ detection.

A Cu_2_O@cMWCNTs/ITO electrochemical sensing platform was fabricated through a sequential drop-casting and electrodeposition strategy for NO_3_^−^ determination. The preparation procedure and sensing principle were illustrated in Figure 1. ITO is widely employed for the construction of electrochemical sensors owing to its high electrical conductivity, optical transparency, low cost, and ease of surface modification (Li C. et al., 2026; Li J. et al., 2026; Lin et al., 2026a; Lin et al., 2026b; Shi et al., 2025). In this work, cMWCNTs were first dispersed in ultrapure water by ultrasonication to obtain a stable and homogeneous suspension. Benefiting from abundant oxygen-containing groups, cMWCNTs exhibited good dispersibility in aqueous media and could be uniformly deposited onto the ITO surface by a simple drop-casting process.

After drying, an interconnected cMWCNT film could be formed on the electrode, providing a three-dimensional conductive network. The cMWCNT layer was expected to contribute to the sensing interface in several aspects. First, the intrinsic conductivity of carbon nanotubes facilitated interfacial electron transport at ITO electrode. Second, the entangled nanotube network increased the accessible electroactive surface area and provides abundant nucleation sites for the subsequent growth of Cu_2_O nanoparticles. Third, surface functional groups on cMWCNTs can strengthen the interaction between the carbon framework and metal oxide species, thereby favoring the uniform deposition and stable immobilization of Cu_2_O. Electrodeposition is a facile and controllable strategy for fabricating nanocatalyst modified electrodes as it enables direct growth or deposition of catalytic materials on conductive substrates (Li et al., 2024; Lu et al., 2026a; Lu et al., 2026b; Wu et al., 2025; Yan et al., 2026). Various functional materials, including noble-metal nanoparticles (Pei et al., 2025; Wu L. et al., 2026; Zhang H. et al., 2023; Zhou et al., 2024), metal oxides (Fan et al., 2025a; Fan et al., 2025b; Huang et al., 2026; Jiang et al., 2026), and carbon-based nanomaterials (Fang et al., 2026; Zhang et al., 2025; Zhao et al., 2025), can be deposited or co-deposited to construct high-performance sensing interfaces (An et al., 2024; Wei et al., 2026; Zhou et al., 2025). Using this method, Cu_2_O nanoparticles were then grown *in situ* on cMWCNT-modified ITO electrode in a copper tartrate solution (Anastasiadou et al., 2023). During NO_3_^−^ detection, Cu_2_O served as the electrocatalytic component, promoting the cathodic reduction of NO_3_^−^ and generating a distinguishable reduction current. Meanwhile, cMWCNT network accelerated charge transport and improved mass/electron transfer to the catalytic sites. Therefore, the enhanced NO_3_^−^-sensing performance of Cu_2_O@cMWCNTs/ITO electrode was attributed to the combined contributions of the catalytic activity of Cu_2_O and the conductive scaffold effect of cMWCNTs.

### 3.2 Characterization of cMWCNTs and Cu_2_O@cMWCNTs

**FIGURE 2:**
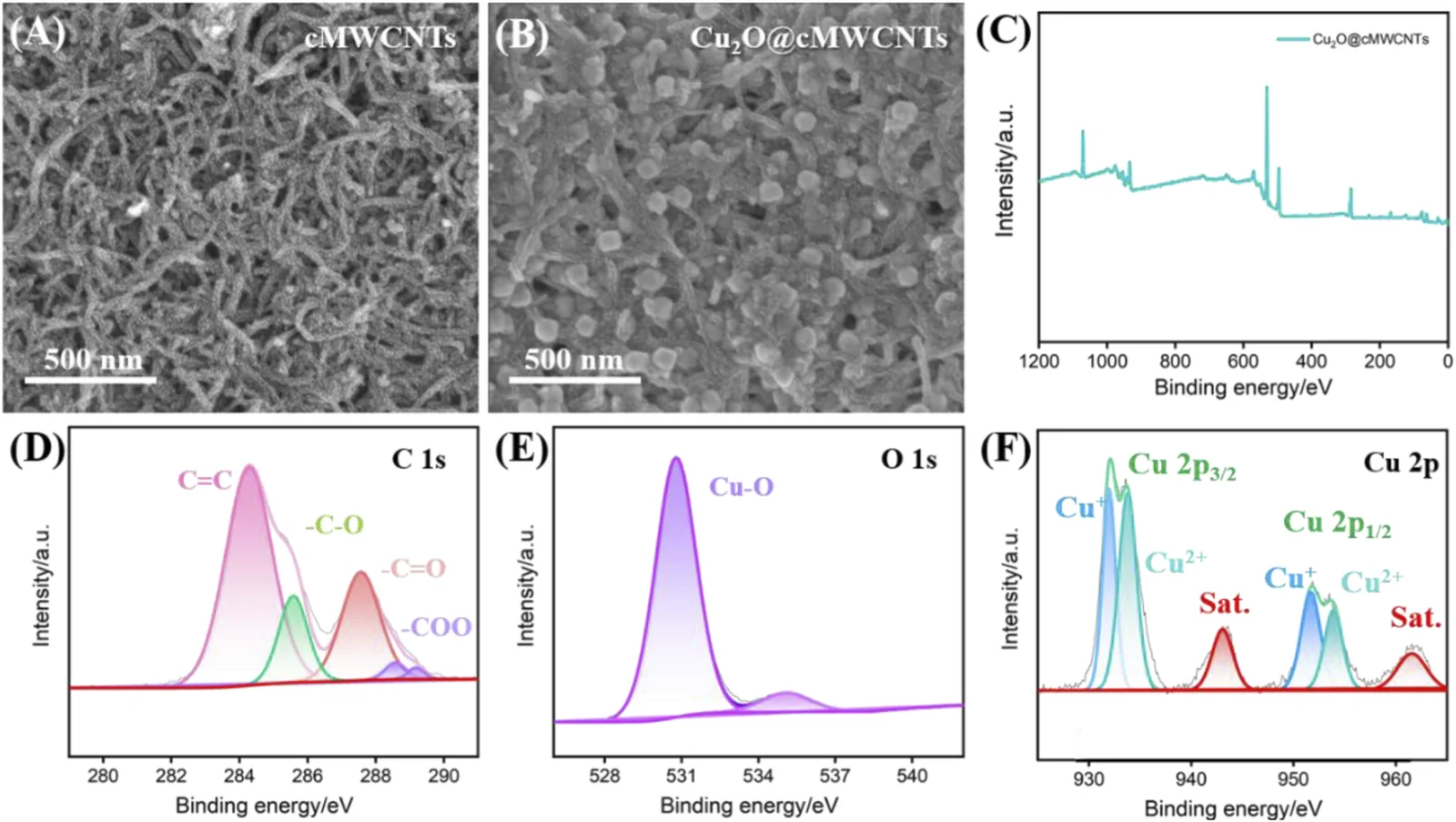
SEM images of **(A)** cMWCNTs and **(B)** Cu_2_O@cMWCNTs. **(C)** XPS survey spectrum of Cu_2_O@cMWCNTs. High-resolution XPS spectra of **(D)** C 1s, **(E)** O 1s, and **(F)** Cu 2p for Cu_2_O@cMWCNTs.

The morphology, elemental composition, and surface chemical states of the prepared materials were investigated by scanning electron microscopy (SEM) and X-ray photoelectron spectroscopy (XPS). As shown, cMWCNTs displayed typical one-dimensional tubular features (Figure 2A). Such an interconnected architecture might be favorable for constructing continuous conductive pathways and offered sufficient spatial sites for Cu_2_O loading. After electrodeposition, numerous nanoparticulate structures appeared on cMWCNT framework (Figure 2B). Compared with pristine cMWCNTs, the particles were distributed throughout the carbon nanotube network. This result suggested that copper oxide species were successfully generated and uniformly distributed on cMWCNT-modified surface.

The XPS survey spectrum of Cu_2_O@cMWCNTs (Figure 2C) showed C, O, and Cu signals, confirming the coexistence of the carbon nanotube support and copper oxide species. In the high-resolution C 1s spectrum (Figure 2D), the peak at ∼284.5 eV was assigned to graphitic sp^2^-hybridized carbon bond (C–C/C=C) (Deng et al., 2023; Gong et al., 2022) of cMWCNTs. The peak near 286.0 eV corresponded to C–O species, while peaks in the range of 287.2–288.0 eV were associated with C=O groups (Li et al., 2022; Xu et al., 2023; Zhu et al., 2025). These groups were originated from the surface functional groups of cMWCNTs. These oxygen-containing groups can improve the wettability of the nanotubes and assist anchoring of Cu_2_O on cMWCNTs during electrodeposition.

The peak at approximately 530.5 eV in O 1s spectrum (Figure 2E) can be assigned to lattice oxygen in Cu_2_O, indicating the formation of Cu–O bonds. Peaks at higher binding energies were generally related to surface hydroxyl groups, adsorbed oxygen species, or adsorbed water molecules. The Cu 2p spectrum (Figure 2F) exhibited characteristic Cu 2p_3/2_ and Cu 2p_1/2_ peaks at ∼932.5 and 952.3 eV, respectively, which were typically attributed to Cu^+^ species, suggesting that Cu_2_O was the dominant copper-containing phase. Weak signals near 934.6 and 954.5 eV, together with satellite featured around 942 and 962 eV, indicating the presence of a small amount of Cu^2+^ species. This phenomenon might arise from slight surface oxidation of Cu_2_O upon exposure to air.

### 3.3 Electrochemical characterization of electrode fabrication process and feasibility for NO_3_^−^ detection

**FIGURE 3:**
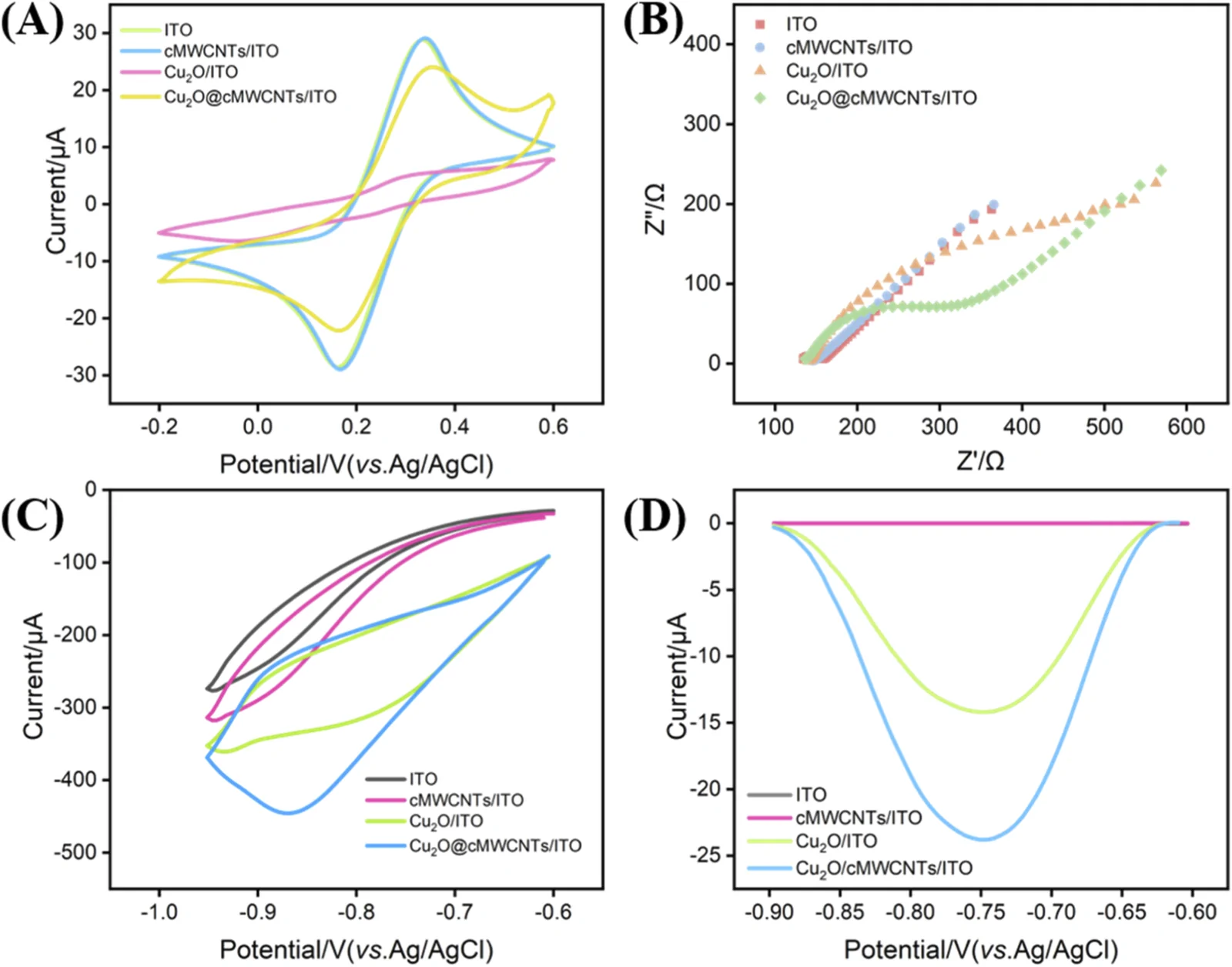
**(A)** CV curves and **(B)** EIS spectra recorded on four different electrodes in KCl solution containing [Fe(CN)_6_]^3-/4-^. **(C)** CV and **(D)** DPV responses of the electrodes in Na_2_SO_4_ solution containing NO_3_^−^ (500 μM). The scan rate for CV measurement was 50 mV/s.

To evaluate the influence of each modification step on interfacial electron-transfer properties, four electrodes, namely, bare ITO, cMWCNTs modified ITO (cMWCNTs/ITO), Cu_2_O deposited on ITO (Cu_2_O/ITO), and Cu_2_O@cMWCNTs/ITO, were examined by cyclic voltammetry (CV) and electrochemical impedance spectroscopy (EIS) using ferri/ferrocyanide ([Fe(CN)_6_]^3-/4-^) redox couple. Bare ITO exhibited a pair of well-defined redox peaks (Figure 3A). After modification with cMWCNTs, the peak current of cMWCNTs/ITO changed only slightly compared with that of bare ITO. This behavior can be explained by the combined effects of improved conductivity and electrostatic interaction. Although the conductive nanotube network tends to enhance electron transfer and increase the effective surface area, the carboxyl groups on cMWCNTs can be deprotonated under near-neutral conditions, imparting a negatively charged interface. Consequently, the anionic [Fe(CN)_6_]^3-/4-^ probe may experience electrostatic repulsion, which partly suppressed the expected increase in peak current. In contrast, after Cu_2_O was deposited directly on ITO, the obtained Cu_2_O/ITO electrode showed a remarkably decreased redox current, suggesting that Cu_2_O layer hindered electron transfer. In contrast, Cu_2_O@cMWCNTs/ITO electrode showed a higher current, demonstrating that cMWCNT layer improved the conductivity of the Cu_2_O-modified interface. This conclusion was further supported by EIS measurements (Figure 3B). The charge-transfer resistance (*R*_ct_) values of ITO, cMWCNTs/ITO, Cu_2_O/ITO, and Cu_2_O@cMWCNTs/ITO were 132, 65, 588, and 220 Ω, respectively. The lower *R*_ct_ of cMWCNTs/ITO (65 Ω) relative to bare ITO (132 Ω) confirmed that the nanotube network facilitated interfacial electron transfer. The substantial increase in *R*_ct_ for Cu_2_O/ITO indicated that Cu_2_O alone partially hindered electron transport. However, the *R*_ct_ of Cu_2_O@cMWCNTs/ITO (220 Ω) was significantly smaller than that of Cu_2_O/ITO (588 Ω), verifying that cMWCNT conductive framework effectively alleviated the charge-transfer limitation caused by Cu_2_O deposition.

The feasibility of the prepared electrodes for NO_3_^−^ sensing was further investigated by CV and DPV. As shown, cMWCNTs/ITO displayed a higher background current in the negative potential region than bare ITO, consistent with the enhanced surface area of the modified interface (Figure 3C). After Cu_2_O loading, both Cu_2_O/ITO and Cu_2_O@cMWCNTs/ITO exhibited obvious cathodic responses, confirming the electrocatalytic activity of Cu_2_O toward NO_3_^−^ reduction. Compared with Cu_2_O/ITO, Cu_2_O@cMWCNTs/ITO electrode produced a much stronger NO_3_^−^ reduction signal. This enhancement might be attributed to the dual role of cMWCNTs. On the one hand, cMWCNTs improved electron-transfer efficiency and provide a high-surface-area support for Cu_2_O growth, thereby increasing the accessible catalytic sites. DPV measurements (Figure 3D) further revealed that electrodes containing Cu_2_O generated distinct NO_3_^−^ responses, while Cu_2_O@cMWCNTs/ITO exhibited the highest peak current among the tested electrodes. These results confirmed the combined contributions between Cu_2_O and cMWCNTs. Cu_2_O acted as the catalyst for NO_3_^−^ electroreduction, whereas cMWCNTs enhanced the sensing performance by improving conductivity, increasing interfacial area, and promoting Cu_2_O dispersion.

**FIGURE 4:**
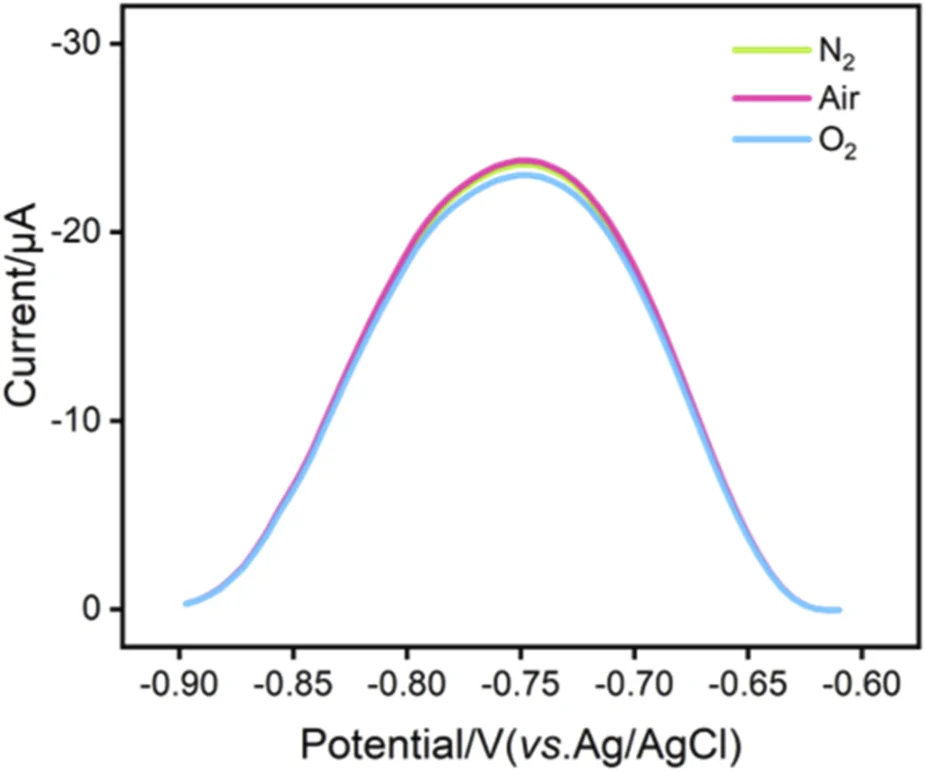
DPV responses of Cu_2_O@cMWCNTs/ITO electrode in 0.1 M Na_2_SO_4_ solution containing 500 μM NO_3_^−^ under different atmospheres (N_2_, air, and O_2_).

The reliability of the Cu_2_O@cMWCNTs/ITO electrode for NO_3_^−^ detection was further assessed by examining its response under different atmospheric conditions, its tolerance toward common coexisting ions, and the reproducibility of independently fabricated electrodes. The influence of dissolved oxygen was first examined because oxygen reduction might interfere with cathodic electrochemical measurements. DPV responses were recorded in nitrate-containing electrolyte under N_2_, air, and O_2_ atmospheres. Three voltammetric curves were very similar, indicating that NO_3_^−^ signal was not affected by the oxygen content in solution (Figure 4). Thus, NO_3_^−^ can be measured under ambient conditions without nitrogen purging, which simplifies the detection procedure.

### 3.4 Optimization of experimental conditions

**FIGURE 5:**
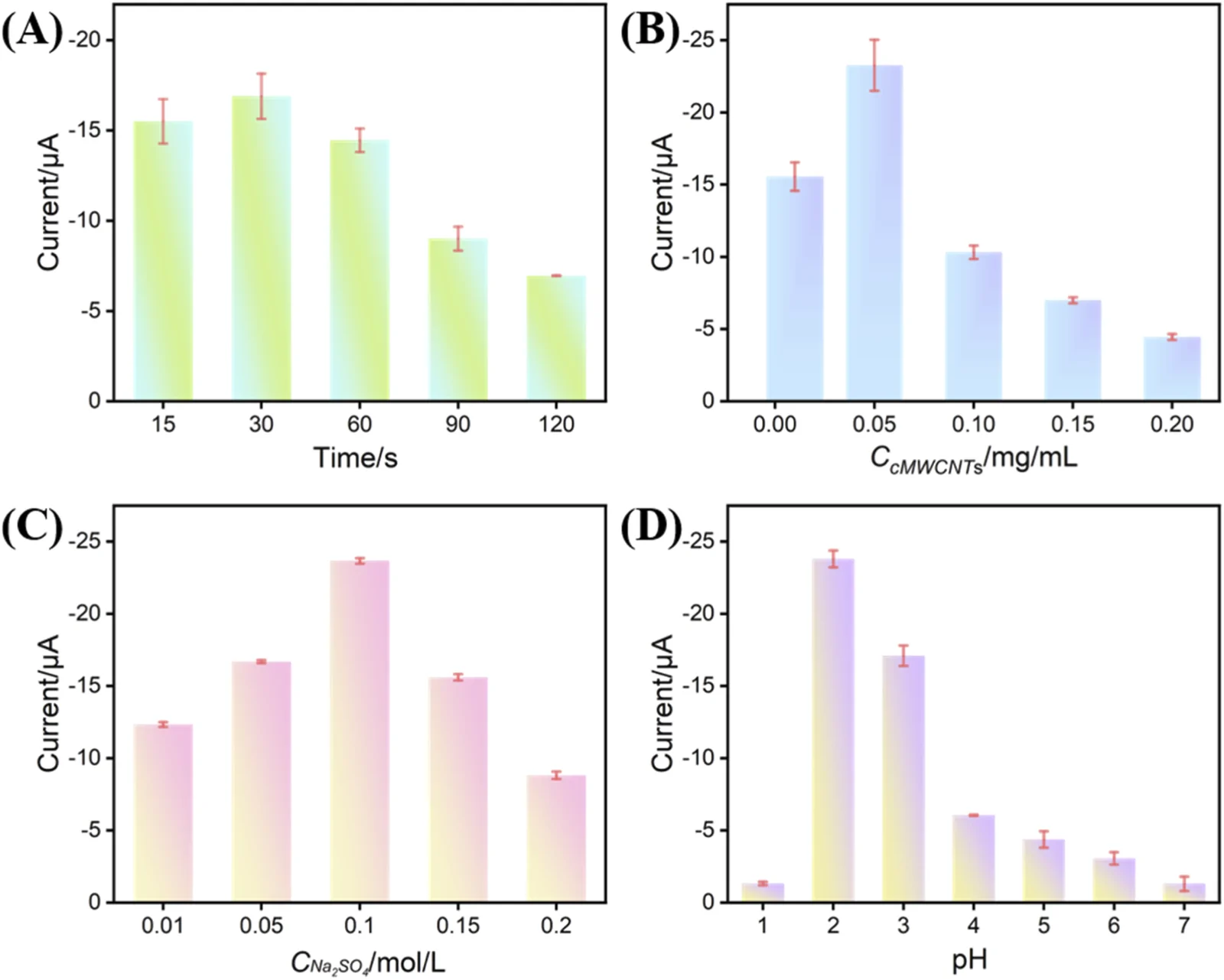
Effects of **(A)** Cu_2_O electrodeposition time, **(B)** cMWCNT concentration, **(C)** Na_2_SO_4_ concentration, and **(D)** solution pH on electrochemical detection of NO_3_^−^. Three measurements were used to calculate the error bars.

Several experimental parameters were optimized to maximize the analytical performance of the Cu_2_O@cMWCNTs/ITO electrode, including Cu_2_O deposition time, cMWCNT concentration, electrolyte Na_2_SO_4_ concentration, and detection pH. The effect of Cu_2_O deposition time on NO_3_^−^ response was shown in Figure 5A. The NO_3_^−^ reduction current increased when Cu_2_O deposition time was extended from 15 to 30 s. This indicated that more Cu_2_O active sites were introduced during the early stage of deposition. However, excessive deposition might produce a thicker oxide layer and cause particle aggregation, which can block active sites and slow down mass transport, leading to reduced current signal. Thus, Cu_2_O deposition time was selected as 30 s. The cMWCNT concentration also influenced the sensor response (Figure 5B). When the cMWCNT concentration increased from 0 to 0.05 mg mL^−1^, NO_3_^−^ signal increased obviously. However, when the concentration was further increased to 0.20 mg mL^−1^, the current response decreased. Thus, moderate amount of cMWCNTs can improve surface conductivity and provide anchoring sites for Cu_2_O growth. Thick nanotube layer might reduce film permeability and increase the diffusion distance of NO_3_^−^. Thus, 0.05 mg mL^−1^ cMWCNTs were used.

The influence of supporting electrolyte concentration was investigated by varying Na_2_SO_4_ concentration from 0.01 to 0.20 M (Figure 5C). The current response increased with increasing Na_2_SO_4_ concentration from 0.01 to 0.10 M. When the concentration reached 0.20 M, the current decreased, which may be associated with excessive ionic strength and less favorable NO_3_^−^ transport near the electrode surface. The cathodic current increased as the pH decreased from 7 to 2, indicating that acidic conditions promoted NO_3_^−^ electroreduction (Figure 5D). This behavior resulted from the phenomenon that NO_3_^−^ reduction involved proton-coupled electron-transfer steps. However, at pH 1, the current nearly disappeared. Under such strongly acidic conditions, Cu_2_O might become unstable, and hydrogen evolution might compete with NO_3_^−^ reduction. Thus, 0.10 M Na_2_SO_4_ with adjusted pH 2 was chosen.

### 3.5 Performance for quantitative determination of NO_3_^−^

**FIGURE 6:**
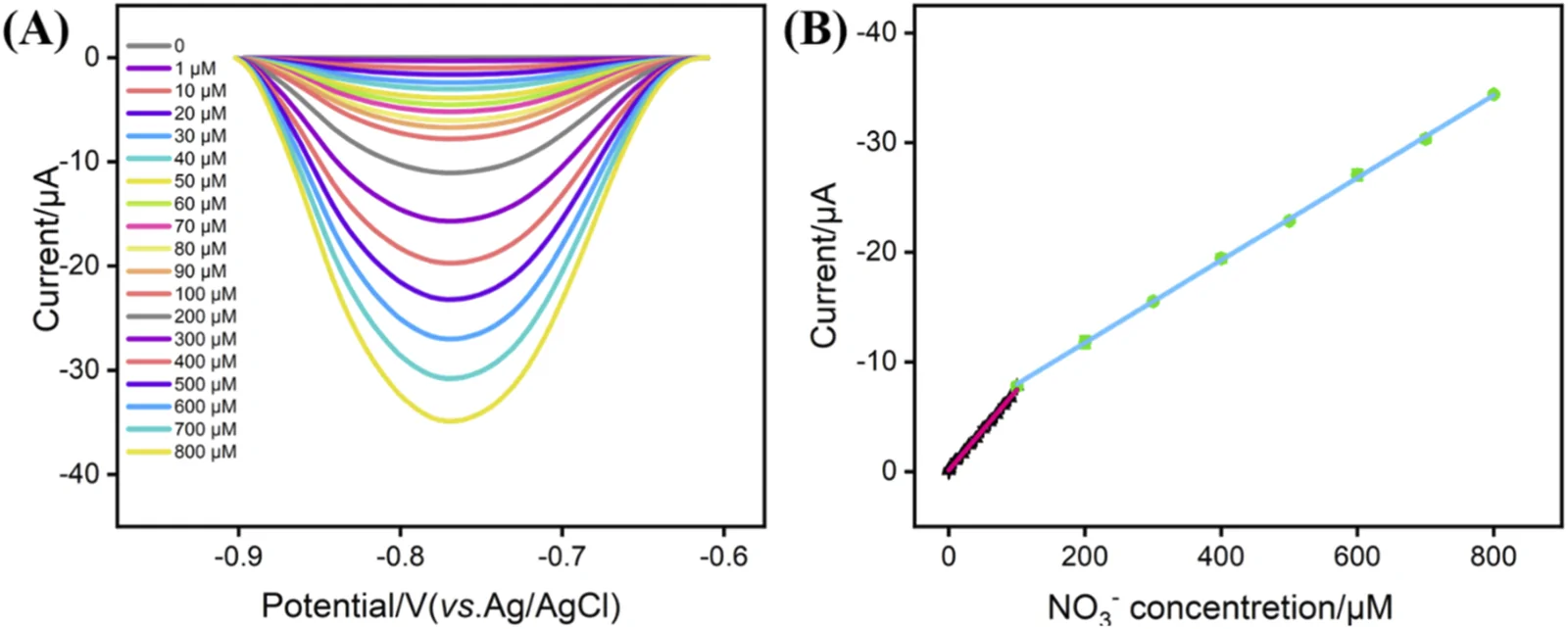
**(A)** DPV responses of Cu_2_O@cMWCNTs/ITO electrode in 0.1 M Na_2_SO_4_ containing different concentrations of NO_3_^−^. **(B)** Calibration plots of DPV peak current versus NO_3_^−^ concentration. Three measurements were used to calculate the error bars.

DPV was employed to evaluate the quantitative sensing performance of Cu_2_O@cMWCNTs/ITO electrode toward NO_3_^−^. Figure 6A showed DPV curves obtained in electrolyte containing different concentrations of NO_3_^−^ from 0 to 800 μM. With increasing NO_3_^−^ concentration, the cathodic peak current in the corresponding negative potential region increased continuously, demonstrating that the composite electrode was sensitive to changes in NO_3_^−^ concentration (Figure 6A). As summarized in Figure 6B, DPV peak current exhibited two well-defined linear relationships with NO_3_^−^ concentration over the ranges of 1–100 and 100–800 μM. In low concentration range, the calibration equation between the peak current (*I*) and NO_3_^−^ concentration (*C*) was *I*_1_ = −0.072 (±0.00046) *C* (μM) − 0.20 (±0.019) with a correlation coefficient (*R* ^2^) of 0.999. For the higher concentration interval from 100 to 800 μM, the linear regression equation was *I*_2_ = −0.038 (±0.00018)*C* (μM) − 4.2 (±0.060), *R* ^2^ = 0.999. The lower sensitivity at higher nitrate concentrations might result from progressive occupation of the accessible catalytic sites. The limit of detection (LOD) was calculated according to LOD = 3SD/*k,* where SD was the standard deviation of the blank signal and *k* is the slope of the low-concentration calibration plot. The LOD was determined to be 0.79 μM. These results demonstrated that Cu_2_O@cMWCNTs/ITO electrode provided a wide detection range and low limit of detection.

### 3.6 Anti-interference performance and reproducibility

**FIGURE 7:**
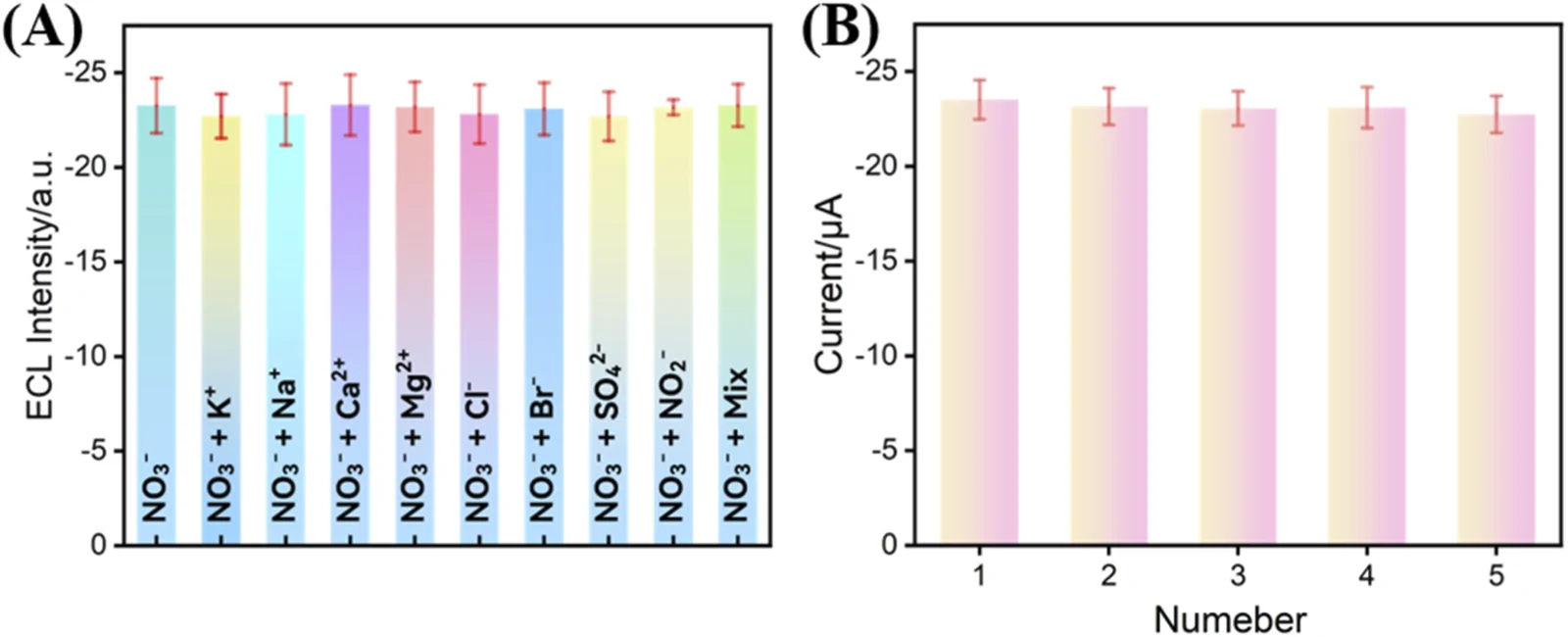
**(A)** Anti-interference performance of Cu_2_O@cMWCNTs/ITO electrode for NO_3_^−^ detection in presence of co-existing ions including K^+^ (0.1 M), Na^+^ (0.1 M), Ca^2+^ (0.1 M), Mg^2+^ (0.1 M), Cl^−^ (0.1 M), Br^−^ (1 mM), SO_4_^2-^ (0.1 M), NO_2_^−^ (1 mM), and their mixture; **(B)** Reproducibility of DPV responses of five independently prepared Cu_2_O@cMWCNTs/ITO electrodes in 0.1 M Na_2_SO_4_ solution containing 500 μM NO_3_^−^. Three measurements were used to calculate the error bars.

The selectivity of the sensor was investigated by introducing common ions, including K^+^, Na^+^, Ca^2+^, Mg^2+^, Cl^−^, Br^−^, SO_4_^2−^, NO_2_^−^, and a mixture of these species (Figure 7A). DPV response toward NO_3_^−^ remained almost unchanged after the addition of these potentially interfering ions, indicating good anti-interference capability. Common cations such as K^+^, Na^+^, Ca^2+^, Mg^2+^, as well as anions such as Cl^−^ and SO_4_^2−^, generally did not exhibit pronounced electrochemical activity within the selected potential range and therefore did not directly affect the nitrate reduction peak. Although NO_2_^−^ was an electroactive nitrogen oxyanion, its reaction potential and interfacial reaction pathway differed from those of NO_3_^−^ under the present experimental conditions, resulting in limited interference. The stable response observed in the mixed-ion system further confirmed the applicability of the Cu_2_O@cMWCNTs/ITO electrode to NO_3_^−^ detection in complex aqueous matrix.

Reproducibility was evaluated using five independently prepared Cu_2_O@cMWCNTs/ITO electrodes. These electrodes produced highly consistent DPV responses toward NO_3_^−^ under identical conditions, with a relative standard deviation (RSD) of 2.3% (Figure 7B), indicating high reproducibility for electrode fabrication. The fabricated sensors were stored at 4 °C and measured the nitrate (500 μM) detection signals of the electrodes at different storage intervals. The results show that after 10 days of storage, the electrodes retained 92.3% of the initial responsive signal, demonstrating that the electrodes exhibited good storage stability.

### 3.7 Determination of NO_3_^−^ in seawater samples

To evaluate the practical feasibility of Cu_2_O@cMWCNTs/ITO sensor in complex environmental samples, NO_3_^−^ in seawater was analyzed. Seawater contained high concentrations of inorganic salts and diverse matrix components, providing a representative medium for assessing the applicability of the sensor to real water analysis. Prior to measurement, the pH of the seawater sample was adjusted to 2 to match the optimized detection condition. DPV measurements were then performed directly using Cu_2_O@cMWCNTs/ITO electrode, and the accuracy of the method was assessed by the standard addition method. As listed in Table 1, the NO_3_^−^ concentration in the original seawater sample was 9.05 μM. After the addition of NO_3_^−^ standards, the recoveries were between 104% and 109%, with RSD values from 2.5% to 4.4%. These results indicated that the proposed sensor can determine NO_3_^−^ in seawater with acceptable accuracy and repeatability, suggesting that the high-salinity matrix did not cause serious interference using the fabricated sensor.

**TABLE 1:** Determination of NO_3_^−^ in seawater using Cu_2_O@cMWCNTs/ITO sensor.

Sample	Added/(μM)	Found/(μM)	RSD/(%, n = 3)	Recovery/(%)
Seawater	—	9.05	—	—
	1.00	10.1	3.8	105
	10.0	19.9	2.5	109
	100	113	4.4	104

## 4 Conclusion

In this work, Cu_2_O@cMWCNTs/ITO electrochemical sensing interface was fabricated on ITO glass through a combined drop-casting and electrodeposition strategy and applied for sensitive determination of NO_3_^−^. Cu_2_O@cMWCNTs modified ITO electrode was prepared by combining cMWCNT drop-casting with Cu_2_O electrodeposition. The cMWCNT layer formed a conductive network on ITO surface and provided anchoring sites for Cu_2_O nanoparticle growth. Compared with Cu_2_O deposited directly on ITO, Cu_2_O@cMWCNTs composite interface showed improved charge-transfer behavior and higher NO_3_^−^ reduction current. The sensor can achieve sensitive NO_3_^−^ detection. Interference tests showed that common inorganic ions including NO_2_^−^ had limited effects on NO_3_^−^ determination. The sensor had good fabrication reproducibility. In real seawater samples, NO_3_^−^ can be detected using standard addition method with satisfactory recoveries. This Cu_2_O@cMWCNTs/ITO platform offered simple fabrication, sensitive detection and good selectivity, providing an effective strategy for electrochemical detection of NO_3_^−^ in complex environments. The present platform used an ITO substrate and manual drop-casting of cMWCNTs. Future work should evaluate scalable and portable electrode formats, such as screen-printed electrodes. Additionally, broad validation using seawater samples with different salinities, organic-matter contents, and nitrate concentrations should be investigated in the future.

## Data Availability

The original contributions presented in the study are included in the article/Supplementary Material, further inquiries can be directed to the corresponding authors.
