# Supplementary material for: Cu2O nanoparticles electrodeposited on carboxylated multiwalled carbon nanotubes for sensitive electrochemical detection of nitrate in seawater
Source: Front Chem. 2026 Aug 31;14:1947669. doi: 10.3389/fchem.2026.1947669 (PMC13572416; doi:10.3389/fchem.2026.1947669)
Supplement: Supplementary file 1 [file Supplementaryfile1.docx]

**Supplementary Materials**

**1 Chemicals and materials**

Carboxylated multi-walled carbon nanotubes (cMWCNTs, 95%), potassium ferricyanide (K_3_[Fe(CN)_6_], 99.5%), potassium ferrocyanide (K_4_[Fe(CN)_6_], 99%), sodium hydroxide (NaOH, 98%), sodium chloride (NaCl, 99%), sod

ium sulfate (Na_2_SO_4_, 99%), calcium chloride (CaCl_2_, 97%), magnesium chloride (MgCl_2_, 98%), sodium nitrite (NaNO_2_, 98%), L-tartaric acid (99%), and copper sulfate pentahydrate (CuSO_4_·5H_2_O, 99%) were purchased from Aladdin Biochemical Technology Co., Ltd. (Shanghai, China). Potassium nitrate (KNO_3_, 98%), acetone, anhydrous ethanol (99.8%), and concentrated hydrochloric acid (HCl, 38%) were obtained from Shuanglin Chemical Reagent Co., Ltd. (Hangzhou, China). Unless otherwise stated, all reagents were of analytical grade and were used as received without further purification. Ultrapure water with a resistivity of 18.2 MΩ cm was used throughout all experiments. Indium tin oxide (ITO) coated glass was purchased from Zhuhai Kaivo Optoelectronic Technology Co., Ltd. (Guangdong, China). Prior to use, ITO were cut into the desired dimensions and sequentially ultrasonicated in acetone, anhydrous ethanol, and ultrapure water to remove organic contaminants and particulate impurities. The cleaned ITO electrodes were then immersed in 1 M NaOH solution overnight to further eliminate residual surface contaminants and improve surface hydrophilicity. After treatment, the electrodes were thoroughly rinsed with ultrapure water and dried under a nitrogen stream before use.

**2 Measurements and instrumentations**

The surface morphologies of cMWCNTs and Cu_2_O@cMWCNTs composites were characterized by scanning electron microscopy (SEM, Zeiss, Germany). X-ray photoelectron spectroscopy (XPS) was used to analyze the elemental composition and surface chemical states of the prepared materials. Cyclic voltammetry (CV), differential pulse voltammetry (DPV), and electrochemical impedance spectroscopy (EIS). All electrochemical experiments were conducted in a conventional three-electrode configuration, consisting of an Ag/AgCl reference electrode, a platinum wire or platinum sheet counter electrode, and a bare or modified ITO electrode as the working electrode. CV measurements were performed at a scan rate of 50 mV s^-1^. For interface investigation, (EIS) measurements were performed using an Autolab electrochemical workstation (PGSTAT302N, Switzerland).
